# Addressing Capacity Constraints of Rural Local Health Departments to Support Climate Change Adaptation: Action Is Needed Now

**DOI:** 10.3390/ijerph192013651

**Published:** 2022-10-21

**Authors:** Matthew V. Vo, Kristie L. Ebi, Tania M. Busch Isaksen, Jeremy J. Hess, Nicole A. Errett

**Affiliations:** 1 Public Health–Global Health, University of Washington School of Public Health, Seattle, WA 98195, USA; 2 Department of Environmental and Occupational Health Sciences, University of Washington School of Public Health, Seattle, WA 98105, USA; 3 Department of Global Health, University of Washington School of Public Health, Seattle, WA 98195, USA; 4 Center for Health and the Global Environment (CHanGE), University of Washington School of Public Health, Seattle, WA 98195, USA; 5 Department of Emergency Medicine, University of Washington School of Medicine, Seattle, WA 98195, USA

**Keywords:** climate change, adaptation, public health, practice, rural

## Abstract

Looming climate change health impacts among rural communities will require a robust health system response. To reduce health inequities and promote climate justice, rural local health departments (LHDs) must be adequately resourced and supported to engage in climate change mitigation and adaptation policy and program development and implementation. In the United States, small local tax bases, overreliance on revenue from fee-based services, and limited federal funding to support climate change and health programming, have left rural LHDs with limited and inflexible human, financial, and political capital to support engagement in local climate change activities. Because of the urgent demands stemming from climate change, additional investments and supports are needed to rapidly build the capacity and capability of rural LHDs. Federal and state approaches to public health funding should consider the unique climate change and health risks of rural communities. Further, cross-jurisdictional shared service arrangements and state-level support to build rural LHDs’ technical capacity, and research on local impacts and culturally appropriate solutions, must be prioritized.

## 1. Introduction

Climate change is leading to direct and indirect health impacts, with rural communities at the front lines. Extreme events such as heat waves, wildfires, and drought, projected to increase in frequency, duration, and magnitude in coming years [1,2], alongside more gradual changes (e.g., sea level rise, changes in the geographic range, and seasonality of disease-transmitting vectors), threaten nature benefits for health (e.g., provisioning services such as agriculture, range/pasturing, forestry, and mining [3,4]) that rural communities rely on for their economic stability, as well as social-ecological connections vital to health and well-being [5]. Indirect health impacts, e.g., through contaminated water and food supplies [6,7], and more direct physical (e.g., injury) and mental health (e.g., anxiety, depression) impacts [8], have the potential to overwhelm the limited healthcare and public health capabilities of more remote areas [2,9].

Community-level adaptation to the health impacts of climate change depends on a robust health system. However, the limited capacity of rural health systems across the United States may preclude their ability to adequately prepare for and implement policies and programs to effectively manage adaptation to climate change’s harmful consequences compared to their urban counterparts.

In the United States, a local health department (LHDs) is “an administrative or service unit of local or state government, concerned with health, and carrying some responsibility for the health of a jurisdiction smaller than the state” [10]. Across the country, there are approximately 2800 LHDs with diverse organizational structures, administrative structures, and technical capabilities. All support the provision of “foundational public health services” to their communities that are “the minimum package of public health services that no jurisdiction can be without” [10,11]. These include five foundational areas (communicable disease control; environmental health; maternal, child, and family health; and access to and linkage with clinical care) supported by eight foundational capabilities (assessment and surveillance; community partnership development; equipment; organizational competencies; policy development and support; accountability and performance management; emergency preparedness and response; and communications) [11].

Here, we describe challenges unique to rural LHDs when preparing for and responding to the health impacts of climate change and propose broad areas of focus to support their relevant capacity development. While our focus here is on the United States, additional exploration and comparison of issues germane to rural communities in other countries and contexts is necessary.

## 2. Challenges of Rural LHDs in Managing the Health Risks of Climate Change

Rural LHDs’ capabilities are limited as compared to their urban counterparts due to their financial and human resources constraints, and their roles as a safety net healthcare provider in communities with limited health care alternatives [12,13].

Funding: As evidenced throughout the COVID-19 pandemic, chronic federal and state level disinvestment has weakened the U.S. health system, and rural LHDs are no exception. In fact, small local tax bases and LHD overreliance on fee-for-service revenue streams (e.g., regulatory permitting fees, Medicaid, Medicare, health insurance, etc.) [10] exacerbate the situation as they leave rural LHDs with limited capacity to provide complete population health services even on a “good day” [14,15]. At the same time, the structure of federal funding to support climate change and health programming has systematically reduced opportunities for meaningful engagement by rural LHDs. Specifically, the Centers for Disease Control and Prevention (CDC) have twice funded the Building Resilience Against Climate Effects (BRACE) program to promote climate change and health capacity among state and local jurisdictions. The BRACE program includes a five-step process to help departments prepare for and respond to the health impacts of climate change: (1) anticipate climate impacts and assess vulnerability; (2) project the disease burden; (3) assess public health interventions; (4) develop a climate and health adaptation plan; and (5) evaluate impact and improve quality of activities [16]. While these funds are theoretically open to any LHD, awards are prioritized based on previous BRACE framework implementation, population size, and other factors [17]. As rural LHDs have less baseline capacity (e.g., because of their smaller populations, they have less funding and ability to recruit and retain staff, particularly specialized staff) [10,13], they may be less competitive than their state and urban counterparts, if they have the capacity to submit an application at all. As a result, although the majority of funded recipients were state-level public health agencies that include rural jurisdictions within their service areas, no awards were made directly to rural LHDs [18].

Human Resources: As noted, rural LHDs’ have challenges recruiting and retaining a workforce adequate in size and capability to meet the diverse needs of their populations [10,13,15]. Limited access to healthcare in many rural communities, as well as rural LHDs’ significant reliance on associated revenue [10], mean that rural LHD staff must focus on providing stop-gap, basic clinical health services [14,19]. Similarly, the need for professional staff to work as generalists covering a variety of services driven by permitting or other fee-for-service activities, leaves rural LHDs less equipped to support specialty-focused activities. Furthermore, limited staff capacity and competing priorities often precludes “optional” activities, such as climate change adaptation, which diminishes rural LHDs’ ability to acquire additional resources to increase adaptation efforts. Limited staffing further reduces rural LHDs capacity to engage in competitive grant writing [15,20].

Political barriers: Concomitantly, rural LHDs may be politically constrained from dedicating resources to climate change adaptation. Elected rural officials in the western United States showed less support for and/or neutrality toward existing federal environmental policies [21], fueled by constituency distrust in government oversight [21]. Sentiments of marginalization may fuel rural distrust of government entities [22], providing unfavorable political environments for LHDs to prioritize climate change adaptation activities [23].

## 3. Proposed Solutions

An equitable response to climate change requires centering the needs and voices of those most vulnerable and with the fewest resources to adapt, including rural communities [24]. Support for rural LHDs is urgently needed, including through direct and sustained funding, additional technical capacity, and rurally focused research to promote evidence-informed decision making and political accountability. Combined, these enhancements can facilitate rural LHDs’ ability to prepare for and respond to the health impacts of changing climate through effective and scalable public health interventions.

Provide Dedicated Funding: Solutions to better provide rural LHDs with the resources required to help their communities adapt to climate change need to reduce systematic barriers and be rurally centered. Dedicated funding to support rural LHD engagement in climate change and health programming is also urgently needed. The Biden–Harris administration’s recent investment of 633 million dollars in “climate-smart and resilient infrastructure [for rural] communities” [25] is one positive precedent. Although these funds primarily support a switch to cleaner energy sources, this effort demonstrates interest in promoting equitable climate change adaptation among rural populations [26]. Similarly, with additional appropriations, CDC could create and resource a rurally tailored version of the BRACE program that accounts for rural LHD capacity constraints and community needs.

Improve Access to Technical Capacity: Collaboration and communication among adjacent rural LHDs can enhance capacity for specialized services. Examples of cross-jurisdictional sharing of public health services include sharing specialized staff (e.g., an epidemiologist) or a service (e.g., laboratory testing) among LHDs [27]. As increasing rural LHD capacity is likely to take years, shared service arrangements can help immediately bridge gaps in expertise necessary to tackle urgent climate and health challenges [19]. Specifically, as neighboring communities are likely to face similar climate change impacts and associated public health consequences, shared service approaches to emergency response planning can expand capacity of adjacent rural LHDs to focus on climate-related issues [28]. For example, two rural New York counties’—Genesee and Orleans—shared service arrangement was reported to result in expansion of capacity to respond to public health emergencies, reduced personnel costs, increased expertise among staff, and a successful application to the CDC for a public health associate (which provided two years of research and analysis that supported both counties) [29].

As very few rural LHDs employ specialized staff (e.g., data scientists) to begin with [10], shared service arrangements among rural LHDs may still not be enough to overcome the environmental health needs of their communities. For example, the limited specialized staff capacity among rural LHDs may limit their capacity to do higher-level analysis and implementation of BRACE, which has been criticized for being overly technical and “academic” [30]. Moreover, shared service arrangements can be administratively burdensome, and rural LHDs may not have the capacity to initiate or sustain them. As such, state-level support and collaboration should also be prioritized to enable rural LHDs to engage in climate change and health activities. The BRACE program, for instance, allows states to partner with counties/LHDs when applying for these funds [17,18].

Build the Evidence Base: Additional research is needed to identify the health impacts of climate change in rural communities and to inform the development and implementation of locally and culturally appropriate solutions. High-priority climate change and health research areas were previously described [31]. With the goal of improving the evidence based in support of rural LHD’s climate change and health programming, specific research questions of interest may include: To what extent has climate change already resulted in illness, injury, and death among rural populations? How will rural health be impacted under various climate change and development scenarios? What is the impact of specific interventions, programs, and policies implemented by rural LHDs on rural health? What human and financial resources do rural LHDs need to effectively respond to climate change?

While specific challenges facing rural LHDs have been described, less information is available about the unique characteristics of rural LHDs that may facilitate their ability to successfully engage in climate change adaptation. For example, health officials working in rural communities may have more familiarity with the climate change impacts experienced by their neighbors, as well as their vulnerability and capability to respond. Further, strong social networks often attributed to rural communities may facilitate rural LHDs’ ability to communicate climate and health risks and motivate community members to prepare [32]. Accordingly, future research should focus explicitly on identifying these strengths.

Implementation of such research must be tailored to the capacity constraints of rural LHDs. For instance, traditional epidemiologic approaches that leverage healthcare utilization or mortality data are hindered by lack of access to data or insufficient available data [33], resulting from lack of healthcare infrastructure in situ and low population density [34]. Alternative approaches, including qualitative methods, may provide more context-specific information about health impacts, and must be coupled with vigorous attempts to advance rural access to public health data and analytical resources.

Community-engaged research, or research that leverages a collaborative approach “with and through groups of people connected by geographic proximity, special interests, or similar situations to address issues affecting the well-being of those people [35],” can ensure integration of local knowledge and worldviews, as well as build trust in communities where political support for climate change adaptation is lacking [36,37]. This trust can strengthen internal advocacy within rural communities, leading to improved local services that enhance environmental protection and climate change disaster response [37,38].

## 4. Conclusions

As rural communities are distinctly affected by the health impacts of climate change, increased investments are needed to build the capacity of rural LHDs to address cascading impacts to the health and wellbeing of their populations. Funding, human resources, and political challenges faced by rural LHDs limit their capacity to tackle this tremendous and fast-growing public health challenge. Thus, rural-focused funding, cross-jurisdictional shared service arrangements, and state-level support to build rural LHD technical capacity, and research on local impacts and culturally appropriate solutions are urgently needed to increase the climate change and health capacities and capabilities of rural LHDs.
